# Disentangling brain activity related to the processing of emotional visual information and emotional arousal

**DOI:** 10.1007/s00429-017-1576-y

**Published:** 2017-11-27

**Authors:** Michał Kuniecki, Kinga Wołoszyn, Aleksandra Domagalik, Joanna Pilarczyk

**Affiliations:** 1 Psychophysiology Laboratory, Institute of Psychology, Jagiellonian University, Ul. Ingardena 6, 30-060 Kraków, Poland; 2 Neurobiology Department, The Małopolska Centre of Biotechnology, Jagiellonian University, Ul. Gronostajowa 7A, 30-387 Kraków, Poland

**Keywords:** Perception, Valence, fMRI, Pupil, Amygdala

## Abstract

Processing of emotional visual information engages cognitive functions and induces arousal. We aimed to examine the modulatory role of emotional valence on brain activations linked to the processing of visual information and those linked to arousal. Participants were scanned and their pupil size was measured while viewing negative and neutral images. The visual noise was added to the images in various proportions to parametrically manipulate the amount of visual information. Pupil size was used as an index of physiological arousal. We show that arousal induced by the negative images, as compared to the neutral ones, is primarily related to greater amygdala activity while increasing visibility of negative content to enhanced activity in the lateral occipital complex (LOC). We argue that more intense visual processing of negative scenes can occur irrespective of the level of arousal. It may suggest that higher areas of the visual stream are fine-tuned to process emotionally relevant objects. Both arousal and processing of emotional visual information modulated activity within the ventromedial prefrontal cortex (vmPFC). Overlapping activations within the vmPFC may reflect the integration of these aspects of emotional processing. Additionally, we show that emotionally-evoked pupil dilations are related to activations in the amygdala, vmPFC, and LOC.

## Introduction

Processing of emotional scenes involves cognitive and emotional components mutually influencing each other (Pessoa 2015). Therefore, the brain underpinnings of this process consist of a variety of interconnected structures. For example, arousal induced by emotionally valenced content is linked to the activity of dedicated structures, which are alleged to prime sensory centers for more elaborate processing of the forthcoming stimuli (Liddell et al. 2005; Sara and Bouret 2012; Markovic et al. 2014). It is, however, unclear if the modulation of information processing in the case of emotional content is solely due to elevated arousal or if certain brain structures are fine-tuned to process emotional visual information irrespective of the level of arousal. Here, we aimed at separating brain activity related to emotional arousal and visual processing of a scene. To this end, we scanned participants using functional magnetic resonance imaging (fMRI) and simultaneously measured pupil size as an index of arousal while presenting negative and neutral images with superimposed visual noise to manipulate the amount of visual information.

In general, the presence of visual noise decreases activity in the higher visual areas, which are involved in object recognition such as the V4, the lateral occipital cortex (LOC), the fusiform face area, and the mid-fusiform area (Grill-Spector et al. 1998; Pratte et al. 2013). By contrast, activity in the lower visual areas is not affected by noise added to natural images (Grill-Spector et al. 1998; Pratte et al. 2013). Overall, the impact of noise on brain activity intensifies along the ventral and dorsal streams of the visual pathway (Tjan et al. 2006), preventing conscious object recognition, which seems to be necessary to evoke an emotional reaction. Schupp et al. (2008) showed that early posterior negativity potential, which is modulated by emotional arousal, differentiates between high and low arousal images only after they exceed the visual noise threshold that allows for object identification. Interestingly, arousing content of an image enables its recognition at a lower threshold of noise (Reinders et al. 2005) and reduces noise perception (Markovic et al. 2014), which are related to greater LOC and amygdala activity (Reinders et al. 2005; Todd et al. 2012b).

Pupil size has been shown to be a convenient index of emotional arousal (Bradley et al. 2008; Henderson et al. 2014; Snowden et al. 2016). In the study by Bradley and colleagues (2008), using emotional visual stimuli, pupillary changes strongly correlated with electrodermal responses, but not with heart rate changes, and thus the authors concluded that pupil size dilation is primarily linked to sympathetic nervous system activity. Moreover, the authors established that pupil dilation reflects arousal rather than a valence dimension of an emotional image (Bradley et al. 2008). A more recent study has shown that emotionally-driven pupil size changes are uninfluenced by various stimulus presentation characteristics, such as time, image repetition, and active or passive viewing mode (Snowden et al. 2016), which provides further evidence for robustness of this measure as an indicator of emotional arousal. Pupil dilation, apart from being a good measure of emotional arousal, is considered to be a particularly useful index of changes in mental effort (Beatty 1982; Kahneman 1973). Indeed, numerous studies employing various tasks demonstrated that pupil size increased as a task difficulty increases (e.g. Alnæs et al. 2014; Karatekin et al. 2004). The brain mechanisms that underlie pupil dilation in response to emotional stimuli were investigated in few studies, differing in aims and methodologies, and did not yield consistent results. Most studies point to the relationship between pupil changes and brain activity primarily within various midline structures linked to emotional response, such as the amygdala, anterior cingulate, insula, thalamus, and locus coeruleus (Sterpenich et al. 2006; Johnstone et al. 2007; Paulus et al. 2015), while one study did not report any correlation (Hermans et al. 2013).

The main goal of our fMRI study was to examine the modulatory role of emotional valence on brain activations related to the processing of visual information and those specifically related to arousal. The amount of available semantic information was parametrically varied by adding visual noise to the negative and neutral images, while pupil size was measured as an indicator of emotional arousal. We expected that if the role of valence in the perception of visual stimuli was limited to modulating arousal, we would observe in the negative condition correlation between pupil size and activity of structures linked to broadly defined emotional processing, such as the amygdala, visual cortex, and prefrontal cortex (Sabatinelli et al. 2011; Garcia–Garcia et al. 2016; Lindquist et al. 2015). If, however, also processing of visual information was modulated by the valence condition, regardless of arousal induced by it, some of these regions would be primarily correlated with the amount of available emotional visual information, but not with pupillary changes. Additionally, we aimed to establish the link between emotionally driven pupil dilations and brain activity.

## Materials and methods

### Participants

Nineteen healthy participants, (9 women) aged 19–29 (*M* = 22.9) with correct vision, no history of neurological disorders, and free from any medical conditions were recruited by community advertisements at the Jagiellonian University Campus. A few days before the experiment, the participants visited an fMRI facility where their ability to see clear images through the NordicNeuroLab goggles was tested. Only participants able to perceive coherent unitary percept through the goggles were invited to take part in the experiment. Upon arrival at the fMRI facility, participants signed an informed consent and a separate agreement to undergo a scanning procedure. The experimental procedure was approved by the ethical committee of the Institute of Psychology at the Jagiellonian University.

### Experimental material

**Fig. 1:**
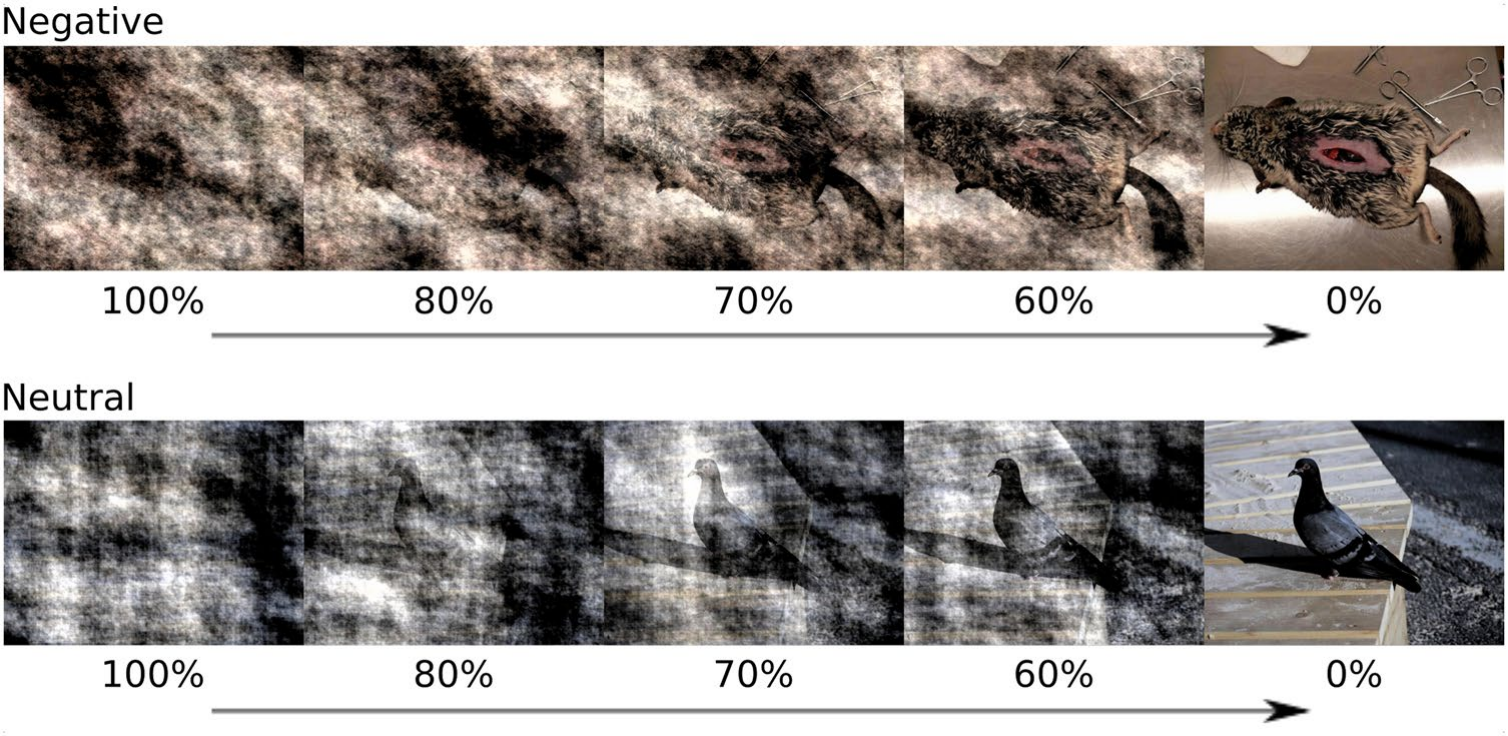
Example of negative and neutral images from NAPS with superimposed noise. Percentage of pink noise content is indicated below each image

A set of 25 negative (valence *M* = 2.62, SD = 0.47; arousal *M* = 6.35, SD = 0.58) and 25 neutral (valence *M* = 5.07, SD = 0.60; arousal *M* = 4.07, SD = 1.15) color images was selected from the International Affective Picture System (IAPS; Lang et al. 2008) and the Nencki Affective Picture System (NAPS; Marchewka et al. 2014). To match images in terms of picture composition, we ensured that the mean size of the key object did not differ between negative and neutral images (10 and 11% of the total image area, respectively; *t*(48) = − 1.0, *p* = .55). The color saturation of the images, measured using the HSV color space, as well as the colorfulness of the images, calculated using Hasler and Süsstrunk (2003) algorithm, did not differ between valence conditions (*t*(48) = 0.84, *p* = .40; *t*(48) = 0.86, *p* = 0.39, respectively). The mean spatial information, calculated as edge energy according to the formula provided by Yu and Winkler (2013) was the same across emotional conditions; *t*(48) = − 0.92, *p* = .36. Moreover, mean energy in the low (< 0.60 cycles per degree) and high (> 1.21 cycles per degree) spatial frequencies, calculated using algorithm by Delplanque et al. (2007), did not differ between emotional and neutral conditions (*t*(48) = − 1.03, *p* = 0.32; *t*(48) = 0.65, *p* = 0.51, respectively). To manipulate signal-to-noise ratio, we mixed pink noise images with original images. Pink noise is obtained by replacing the phase in the Fourier spectrum of an original image with random values between 0 and 2 pi while preserving the amplitudes (Kayser et al. 2006). To each original image, pink noise was added in following proportions: 0, 60, 70, 80, 100% (Fig. 1). Pink noise was generated separately for each proportion and each image. All images were equated for luminance and contrast which were measured as a mean and standard deviation of L* component in L*a*b* color space.

### Procedure

The experiment was divided into five scanning runs (each 10.8 min., 216 volumes). Images, displayed at a resolution of 800 × 600 pixels and spanning 30 horizontal × 23 vertical degrees of the visual field, were presented via the NordicNeuroLab goggles for 5 s (preceded by fixation cross presentation randomly varying between 3 and 6 s) in fixed sequences from pure noise to complete lack of noise. Between sequences the fixation cross was presented for a time window lasting 8 s in average, adjusted so that each sequence lasted precisely 60 s, which ensured that each run accommodated ten sequences. The sequences were shown in a random order, ensuring, however, that in each run a maximum of seven sequences belonged to the same valence category. In total 50 unique sequences were shown during the experiment. To maintain participants’ attention, they were asked to classify one randomly selected image in each sequence as taken outdoors or indoors.

### Data acquisition

Anatomical and functional magnetic resonance imaging (MRI) was performed using a 3T scanner (Magnetom Skyra, Siemens) with the 20-channel head coil. High-resolution, anatomical images were acquired using T1 MPRAGE sequence (sagittal slices; 1 × 1 × 1 mm3 voxel size; TR = 2200 ms, TE = 2.43 ms). Functional images were acquired using an EPI sequence; scan parameters were as follows: TR = 3000 ms, TE = 21 ms, flip angle = 90°, voxel size 2 × 2 × 2.5 mm3, FOV 192 × 192 mm2, GRAPPA acceleration factor 2, phase encoding A > > P. Whole brain image (excluding cerebellum) was covered with 48 axial slices taken in an interleaved fashion. There were five functional runs; acquisition time for each run was 10′48″ (216 volumes). Due to magnetic saturation effects, the first four volumes (dummy scans) of each run were acquired and then discarded by the scanner.

The experimental task was presented through the VisualSystem goggles with binocular eye-tracking cameras (infra-red, 60 Hz) and responses were collected using fiber-optic response button grips (NordicNeuroLab, Bergen, Norway). The ViewPoint infrared EyeTracker (Arrington Research, Scottsdale, AZ, USA) was used for pupil size measurement.

### Pupillary data analysis

First, pupil diameter was converted from arbitrary units to millimeters. Then, a linear interpolation algorithm was applied to estimate pupil diameter during periods when data was lost due to eye-blinks as well as when the pupil diameter was smaller than 1.5 mm or the difference between two subsequent samples was larger than 0.2 mm. The average amount of interpolated data was 19% (SD = 9). One participant was excluded from all further analyses due to 51% pupillary data loss. The pupil dilation index was calculated for each trial and participant by subtracting the mean baseline (1 s before stimulus onset) from mean pupil size in a window from 2 to 5 s following stimulus onset, analogously to Bradley et al. (2008) and Henderson et al. (2014). The resulting pupil dilation index was examined using repeated measures ANOVA with factors of noise level (5 levels) and valence category (negative and neutral). In all cases where the sphericity assumption has been violated, the results are reported with H–F correction. Simple effects were investigated using Bonferroni correction.

### fMRI data analysis

Functional data were analyzed using the FEAT FMRIB Expert Analysis Tool version 6.0 (http://fsl.fmrib.ox.ac.uk/fsl/fslwiki/). The standard preprocessing steps included brain extraction using BET (Smith 2002), slice timing correction, motion correction using MCFLIRT (Jenkinson et al. 2002), spatial smoothing with a Gaussian kernel of full-width at half-maximum of 5 mm, and high-pass temporal filtering with 100 s cut-off. Next, a whole brain General Linear Model (GLM) analysis was conducted for each of five runs separately. Three regressors for each valence category were entered into GLM analysis, that is image onset and two mean centered parametric regressors, one representing noise level and another pupil dilation. Onsets of the indoor/outdoor diagnostic questions were added as a regressor of no interest. For optimal fitting of the hemodynamic response, regressors were convolved with a set of three orthogonalized basis functions obtained using the FLOBS tool within FSL (Woolrich et al. 2004). In this approach, the first basis function represents canonical HRF while two others model temporal and dispersion derivatives. Only the parameter estimates for the first basis function were analyzed on a group level. On a second level, participant’s five runs were combined using fixed-effects. Group level analysis was conducted using a random-effects model with FLAME (Beckmann et al. 2003). Finally, parameter estimates were tested using RANDOMISE, an FSL tool for nonparametric inference based on permutation. We conducted 10K permutations and applied the threshold-free cluster enhancement method for identifying regions of continuous activation (Smith and Nichols 2009). Resulting statistical maps were thresholded at *p* < 0.05 (family-wise error).

## Results

### Pupil size results

**Fig. 2:**
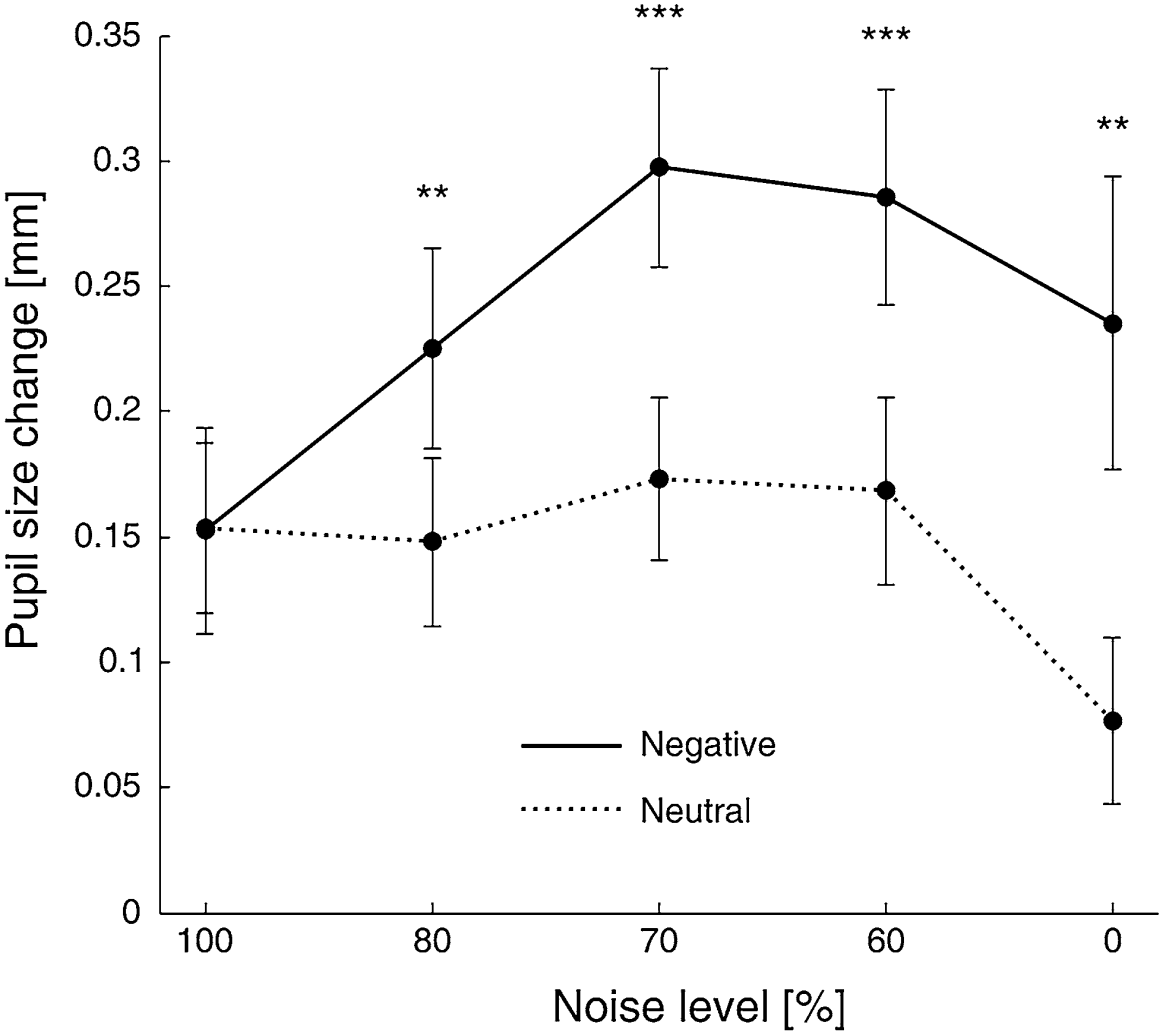
Pupillary changes for negative (solid line) and neutral images (dotted line) in each noise level condition. Asterisks denote significant differences in pairwise comparisons; **p < 0.01, ***p < 0.001. Error bars represent standard error

Pupil dilation was modulated by both valence (*F*(1, 17) = 32.7, *p* < 0.001) and noise level (*F*(4,68) = 5.2, *p* = 0.001). On average, pupil dilation was larger in the negative condition (*M* = 0.24, SD = 0.19) than in the neutral condition (*M* = 0.14, SD = 0.14). Increasing image visibility due to decreasing noise level was parabolically related to changes in pupil dilation as the relationship between noise level and pupil dilation was best explained by the quadratic trend (*F*(1,17) = 10.9, *p* = 0.004). As expected, the interaction of valence and noise also proved to be significant (*F*(4,68) = 5.9, *p* < 0.001). Follow-up pairwise comparisons across valence conditions revealed that pupil dilation was larger in the negative than in the neutral condition at all levels of noise except for the 100% (at *p* < 0.05, see Fig. 2). Pairwise comparisons across noise levels showed that in the neutral condition none of the noise conditions was significantly different from 100% of noise (baseline), while in the negative condition 70 and 80% of noise levels significantly differed from 100% of noise baseline (*p* = 0.005 and *p* = 0.001, respectively). Additionally, linear correlation between noise level and pupil dilation regressors was not significant in either neutral (*r* = − 0.13, *p* = 0.22) or negative (*r* = 0.17, *p* = 0.19) conditions proving that both regressors were explaining unique portions of variance.

### fMRI results

**Fig. 3:**
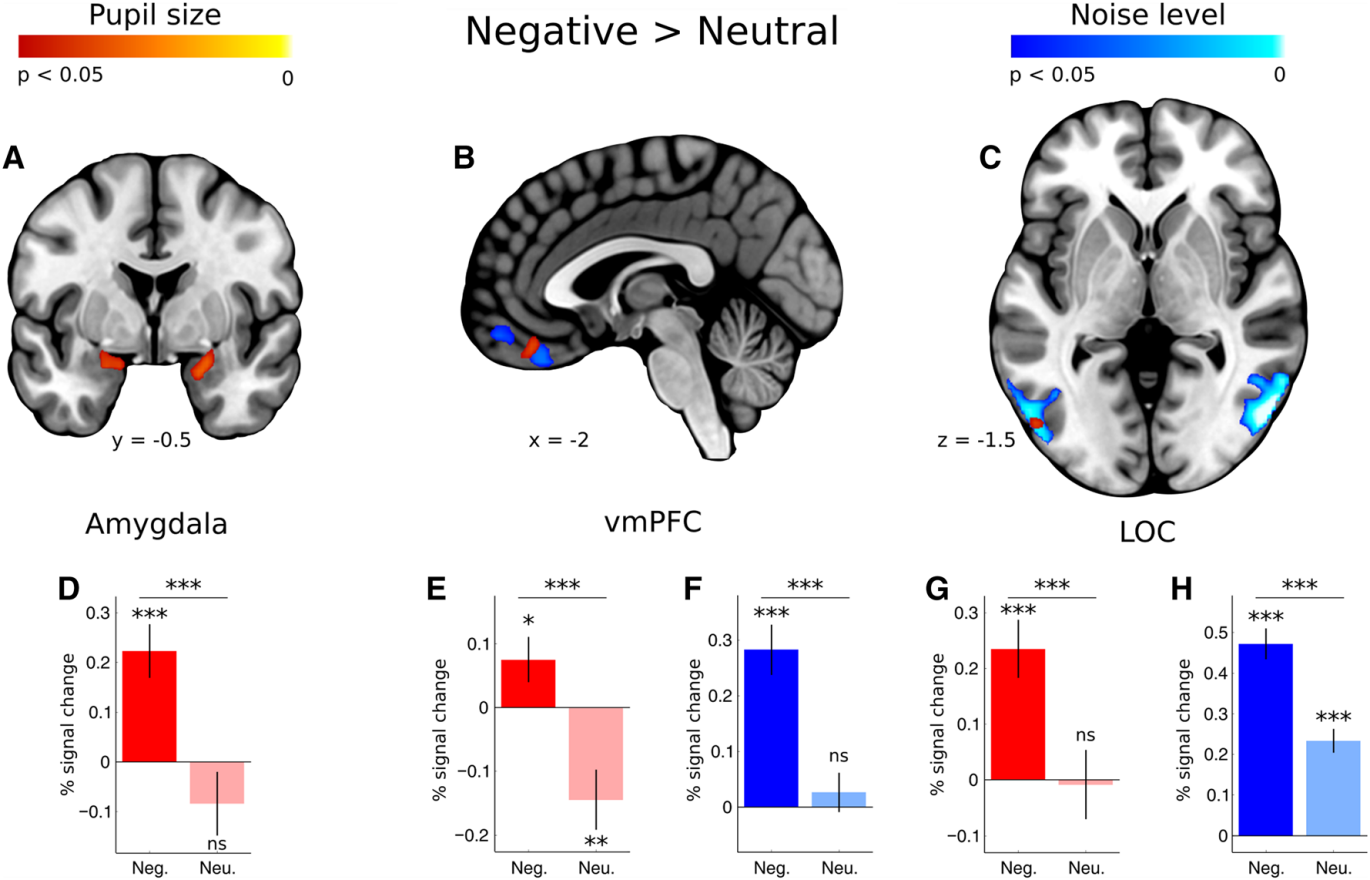
Whole-brain map showing the contrast between negative and neutral images for activations covarying with pupil size (red) and noise level (blue). T-statistical maps were corrected for multiple comparisons with Threshold-Free Cluster Enhancement (TFCE) at *p* < 0.05. **a** Activations in the amygdala, **b** ventromedial prefrontal cortex and **c** lateral occipital cortex. Below, percent signal change as a function of pupil size (red bars) and noise level (blue bars) for negative and neutral images **d** in the amygdala, **e, f** in the vmPFC, **g, h** and in the LOC. Significance of the one-sample *t* test against zero is marked above each bar, while significance of the pairwise comparisons is marked on the top of each graph; *ns* not significant, *p < 0.05, **p < 0.01, ***p < 0.001. Error bars represent standard error

In the negative compared to the neutral condition, the increase in pupil dilation was significantly related to activity within the bilateral amygdala, ventromedial prefrontal cortex (vmPFC), inferior temporal gyrus, middle temporal gyrus and lateral occipital cortex (Table 1; Fig. 3). The direct contrast between the negative and the neutral condition for noise level yielded greater activations in the bilateral occipital cortex and ventromedial prefrontal cortex—the clearer the pictures were, the stronger was the activity in those areas (Table 1; Fig. 3). There were no significant activations in the neutral versus the negative condition.

**Table 1:** fMRI results from the whole brain analysis: brain activity for negative compared to neutral images for pupil dilation and noise level regressors

Brain region	Side	MNI coordinates			*t* value	*p*
		*x*	*y*	*z*		
Pupil dilation						
Amygdala	R	24	2	− 22	5.25	0.023
Amygdala	L	− 18	− 2	− 18	4.66	0.032
Ventromedial prefrontal cortex	L/R	0	40	− 22	4.7	0.035
Inferior temporal gyrus	R	62	− 10	− 34	5.28	0.025
Middle temporal gyrus	L	− 68	− 22	− 14	4.78	0.036
Lateral occipital cortex	L	− 52	− 74	− 2	5.22	0.036
Noise level						
Lateral occipital cortex	R	52	− 72	− 6	5.81	< 0.001
Lateral occipital cortex	L	− 54	− 70	2	6.67	0.002
Ventromedial prefrontal cortex	L	− 2	36	− 26	5.33	0.026

*p* values are corrected for multiple comparisons. All areas have > 20 voxels

*L* left, *R* right

## Discussion

While in most studies, comparison of negative to neutral images yields a pattern of activations encompassing, among others, the lateral occipital cortex, amygdala and prefrontal cortex (for review see: Sabatinelli et al. 2011; Bradley et al. 2014; Garcia–Garcia et al. 2016; Lindquist et al. 2015), here we show that this pattern of activations can be decomposed into two clusters. Valence-related differences in the processing of visual information are linked primarily to activations in the LOC, while emotional arousal is associated with activity in the amygdala. Both processing of emotional visual information and emotional arousal modulate the activity within the ventromedial prefrontal cortex.

Arousal level measured as pupil dilation parabolically increased with the level of scene clarity in the case of negative images and slightly decreased in the case of neutral images. These relationships were not linear, showing that arousal, although not totally independent of the noise level, could not be linearly reduced to it. Importantly, in 100% of noise condition, when the amount of visual information was minimal, the magnitude of pupil dilation was the same for negative and neutral stimuli, constituting a common baseline. Given that pupil dilation can also serve as an index of allocation of attentional resources (e.g. Kahneman 1973), it could be possible that parabolic relation between pupillary changes and levels of scene clarity reflects alterations of cognitive demands. After one randomly chosen image in each sequence, participants were asked to determine whether the image was taken indoors or outdoors. This task was used only to maintain participants’ attention throughout the experimental procedure. However, it cannot be ruled out that attention engaged in this task varied between scene clarity condition, with the greatest engagement in the case of moderate levels of noise (60–80%), in which scene recognition is possible, but not too easy (as in the full clarity condition). Nevertheless, if pupillary changes reflected attention allocation rather than emotional arousal, this pattern of results should be observed irrespective of valence condition. Instead, in the neutral condition there were no significant difference between 100% of noise and other conditions, suggesting that pupillary changes are more likely due to changes in emotional arousal.

Pupil size correlated positively with activity in the amygdala in the negative but not in the neutral condition. This result is consistent with several previous studies, showing that the amygdala is directly related to the arousal level and is involved in the evaluation of emotional or generally significant stimuli (Zald 2003; Sergerie et al. 2008; Sabatinelli et al. 2011; Hayes et al. 2014; Lindquist et al. 2015; Garcia–Garcia et al. 2016). The amygdala activity correlates with various physiological measures of arousal, for example with changes in blood pressure evoked by conditioned stimuli in rats (Shabel and Janak 2009), with skin conductance response in macaque monkeys (Laine et al. 2009) and arousal ratings in humans (Sabatinelli et al. 2005; Wilson-Mendenhall et al. 2013; Bonnet et al. 2015). Our results prove that pupil size is also related to amygdala activity. Such association between amygdala activity and arousal might be due to its bilateral connections with the locus coeruleus, a structure inducing cortical activation. These structures have been shown to coactivate in tasks involving emotional perception or appraisal (Liddell et al. 2005; Sara and Bouret 2012; Markovic et al. 2014; Janak and Tye 2015). Moreover, the large body of amygdalar connections allows it to affect the functioning of both primary sensory areas such as the visual cortex and association areas such as the prefrontal cortex (Amaral et al. 2003; Janak and Tye 2015). Thus, we might speculate that activations related to arousal level in the vmPFC and LOC observed in our study are mediated by the amygdala.

Arousal measured as degree of pupil dilation correlated negatively with activity in the vmPFC in the neutral condition and positively in the negative condition. Out of the few studies investigating brain activations related to pupil diameter (Sterpenich et al. 2006; Johnstone et al. 2007; Hermans et al. 2013; Paulus et al. 2015), none reports activation in the vmPFC. However, since changes of pupil diameter in an emotional context correlate closely with skin conductance (SC; Bradley et al. 2008), our results can be compared with a larger body of brain imaging studies using SC as a measure of physiological arousal. Negative correlations between activity within the vmPFC and SC are usually found when stimuli used in the experiments are non-emotional (Nagai et al. 2004; Fan et al. 2012; Zhang et al. 2014). In contrast, emotional stimuli often yield positive correlations (Critchley et al. 2000; Anders et al. 2004; Williams et al. 2005). Hence, our results fit in with existing evidence insofar as autonomic arousal accompanying presentation of neutral images was correlated negatively with vmPFC activity while arousal caused by negative images was positively correlated. This might reflect the regulatory mechanism by which the vmPFC controls physiological reaction to incoming stimuli akin to Damasio’s somatic marker (Damasio 1996). Accordingly, heightened activity of this prefrontal cortical region might be linked to any shift of arousal from the baseline in response to visual stimuli, regardless of the direction of this change. Precisely, activity in the vmPFC might be responsible for elevating arousal in response to significant stimuli and decreasing it in response to irrelevant stimuli, which results in a positive correlation between vmPFC activity and pupil size in the negative condition and negative correlation in the neutral condition.

Interestingly, vmPFC activity correlated also with the amount of visual information, the correlation being stronger for emotional than for neutral pictures. This result might reflect the processing of emotional information, which is separate from emotional arousal, being rather related to the analysis of the visual content of a scene. With increasing clarity, the context becomes more clear, enabling full evaluation of a scene. This role of the prefrontal cortex is postulated by Barrett and Barr (2009) who argue that the vmPFC acts in concert with visual areas enriching perception with emotional valuation by establishing the relevance of the perceived object to the observer.

Hence, both processing of emotional visual information and emotional arousal activate the same region, the vmPFC. Possibly, populations of neurons engaged in these processes might be located in the same region of the vmPFC, analogous to separate units for detecting positive and negative valence found there in monkeys (Morrison and Salzman 2009) and humans (Chikazoe et al. 2014). Such integrative function of the vmPFC in emotional perception has been postulated by Roy et al. (2012) based on a large review of the literature. He proposed that the vmPFC is responsible for assigning “affective meaning”, by integrating the identity of the incoming stimuli with contextual information. Additionally, thanks to its robust connections with lower brain structures, like the amygdala or the hypothalamus, the vmPFC determines response strategy and orchestrates autonomic and behavioral reactions.

Finally, our results show that increasing amount of visual information is related to enhanced activity within the lateral occipital complex. This corresponds directly with the line of research demonstrating that object identification is linked to LOC activity (Grill-Spector et al. 2001), which varies with objective image clarity (Grill-Spector et al. 1998) and participants’ recognition ability (Grill-Spector et al. 2000). Interestingly, in our study LOC activity accompanying increasing clarity of the images was stronger for negative than for neutral pictures. In general, viewing emotional images is linked to elevated activation in the visual cortex, including the LOC (for reviews see Sabatinelli et al. 2011; Pourtois et al. 2013; Bradley et al. 2014; Markovic et al. 2014). This effect has been conceptualized as reflecting “enhanced sensory processing” (Junghöfer et al. 2006), “motivated attention” (Lang et al. 1998; Bradley et al. 2003) or “affect-biased attention” (Todd et al. 2012a), which presumably involve reentrant connections from the amygdala (Lang et al. 1998; Bradley et al. 2003; Todd et al. 2012a; Pourtois et al. 2013) documented in the macaque (Amaral et al. 2003) and the human brain (Catani et al. 2003; Gschwind et al. 2012). On a functional level, this is further supported by dynamic causal modeling of the MEG data (Rudrauf et al. 2008). This relationship was also confirmed by Todd and colleagues (2012b) who showed that for emotional images activity within the LOC depends on direct causal coupling from the amygdala. Indeed, in our results, the activity of small region within the left LOC was correlated with pupil dilation, like activity in the amygdala, suggesting a functional link between these structures.

However, activation of the much larger region in the LOC was correlated only with scene clarity, which was unrelated to amygdala activity. Thus, LOC activity cannot be entirely attributed to a direct relation between the amygdala and visual areas. We suggest that enhancement of LOC activity in the case of negative images might be due to either increased perceptual clarity of emotional images in noise conditions or greater attention deployment to emotional stimuli. The first hypothesis is supported by the study of Todd et al. (2012b) showing that the emotional images with superimposed noise were perceived as less noisy than the neutral ones, which was related to greater activity in the LOC and amygdala. On the other hand, LOC activity has been shown to be modulated by attention (Murray and Wojciulik 2004). Several studies showed that, in emotional images, semantically relevant objects attract attention more than neutral ones (Humphrey et al. 2012; Niu et al. 2012), an effect which is linearly related to the clarity of an image (Pilarczyk and Kuniecki 2014). Thus, it is possible that the enhanced activity in the LOC in the current study is related to stronger engagement of attention in the case of emotional scenes. Taken together, our results partially add to the evidence supporting the existence of a connection between visual and emotional centers, but they also suggest that enhancement of activity in visual areas, including LOC, during emotional picture viewing might be due to the specificity of affect-related attentional and perceptual processes.

To summarize, our findings demonstrate that emotional arousal and processing of emotionally valenced visual information are linked to independent patterns of brain activations. We argue that the vmPFC, whose activity correlates with both of these components, is engaged in their integration. This substantiates the idea that the vmPFC plays an important role in the unification of emotional experience. Furthermore, our results show that the higher regions of the visual stream are activated more strongly by emotional visual information than by the neutral one regardless of the level of arousal. This observation points to the idea of the fine-tuning of the visual system for processing biologically and behaviorally relevant objects in the environment. Additionally, we established that pupil dilations evoked by emotional stimuli are accompanied by activations in the amygdala, vmPFC, and LOC, advancing the understanding of the brain underpinnings of the arousal-related pupil dilations.
